# Cross-disciplinary awareness of healthcare associated infections (HAIs): insights from a university-wide survey

**DOI:** 10.3389/fmed.2025.1642560

**Published:** 2025-11-10

**Authors:** Arianna Delicati, Dolores Catelan, Beatrice Marcante, Luciana Caenazzo, Pamela Tozzo

**Affiliations:** 1 Legal Medicine Unit, Department of Cardiac, Thoracic, Vascular Sciences and Public Health, University of Padova, Padova, Italy; 2 Department of Pharmaceutical and Pharmacological Sciences, University of Padova, Padova, Italy; 3 Unit of Biostatistics, Epidemiology and Public Health, Department of Cardiac, Thoracic, Vascular Sciences and Public Health, University of Padova, Padova, Italy

**Keywords:** healthcare-associated infections (HAIs), public health, infection prevention, student awareness, educational interventions

## Abstract

**Background:**

Healthcare-associated infections (HAIs) are a major public health problem. Awareness among university students is crucial for prevention. This study analyses the level of knowledge and awareness regarding HAIs and their transmission, identifying factors that influence their risk and importance perception.

**Methods:**

A cross-sectional survey-based study was conducted at the University of Padua, involving students from various academic areas. A total of 1,059 students answered the questions. Inclusion criteria were being enrolled at the University of Padua and having the ability to give voluntary consent to participate. Data were collected through a validated survey with closed and open-ended questions and statistical analyses were performed to investigate the level of knowledge and awareness of HAIs among students aim to provide fundamental for the development of preventive target educational interventions. Moreover, the evaluation of the influences of personal experiences and information on the perception of HAIs and their prevention were also considered.

**Results:**

Students from healthcare areas showed greater awareness of HAIs compared to those from other academic areas. Personal experiences, such as caring for a loved one/relative during hospitalization, increased sensitivity to the topic. The Covid-19 pandemic heightened perceived importance of infection prevention, although knowledge gaps emerged, particularly among non-healthcare students.

**Conclusion:**

Awareness of HAI is widespread, but differences between academic areas persist. Targeted educational strategies and the integration of prevention content into university curricula may enhance overall student engagement, thus contribute to broader prevention efforts.

## 1 Introduction

Healthcare-Associated Infections (HAIs) are infections acquired during the course of healthcare delivery in hospitals, clinics, or other healthcare settings. They represent a significant challenge for healthcare systems worldwide, with highly impactful consequences that include prolonged hospital stays, increased antimicrobial resistance, and higher morbidity and mortality rates (1). Preventing HAIs is essential for improving patient outcomes and ensuring the efficiency of healthcare systems (2). Effective prevention strategies include proper hand hygiene, sterilization of medical devices, and adherence to infection control protocols (3). Healthcare workers (HCWs), patients, and even visitors play a crucial role in controlling the spread of HAIs. Education and training of healthcare staff and the general population are fundamental in raising awareness and promoting preventive behaviors. Reducing the incidence of HAIs not only protects individual patients but also strengthens the overall resilience of healthcare systems, ensuring better resource management and improved public health outcomes (4).

In Italy, recent national surveillance data highlight the significant impact of HAIs within the healthcare context. According to these data, the average prevalence of patients with at least one HAI per hospital was 8.8%, while the overall prevalence across all patients reached 10.2% (5). Among the HAIs recorded, the most frequent types were urinary tract infections, surgical site infections, and lower respiratory tract infections (5). It is important to note that the prevalence rates varied considerably among Italian regions, ranging from 4.17 to 14.14%, emphasizing the heterogeneity of infection control effectiveness across healthcare settings. In particular, data from literature focused on the Veneto Region, where the present study was conducted, are in accordance with these national findings highlighted a prevalence of patients with at least one HAI equal to 7.6% (5, 6). Although these data were collected in an earlier surveillance effort, they remain broadly consistent with the national averages, confirming that the burden of HAIs is significant and relatively homogeneous across Italian regions. Alongside the high prevalence of HAIs, Italy continues to face a serious challenge related to antimicrobial resistance; indeed, national surveillance data indicate widespread resistance among major bacterial pathogens, emphasizing the need for integrated infection prevention and antimicrobial stewardship strategies (5, 7). Collectively, these findings underline the urgent need to strengthen infection prevention and control programs and antimicrobial stewardship initiatives to reduce HAI incidence and improve patient outcomes.

For exogenous infections to occur in healthcare settings, two additional elements are required beyond a susceptible host: a source of infecting microorganisms and a mode of transmission (8). The mechanism of transmission for the various types of HAIs is closely related to the use of invasive medical devices, breaches in skin or mucosal barriers, and deficiency of patients’ immune system. Pathogens can originate from different sources. Infections can be classified as endogenous, originating from microorganisms already present within the hospitalized patient, or more commonly, exogenous, where the source of infection is external to the patient. This mode of transmission can be either direct, when pathogens are transferred directly from the source to the susceptible host, or indirect, when one or more intermediate steps or objects are contaminated before reaching the host (9).

Microorganisms carried by healthcare professionals and visitors play a central role in the transmission of exogenous infections in patients; at the same time, the microbiological characteristics of patients can also lead to both endogenous infections, when the infection affects the patients themselves, and exogenous infections, when their microorganisms are released into the environment and infect other susceptible patients (10–12).

Thus, patients’ microbiota is not the only factor influencing the development of HAIs; HCWs and visitors can also act as asymptomatic carriers, playing a significant role in the large-scale dissemination of HAIs-related bacteria. Their hands, mobile phones, and protective clothing were identified as critical hotspots for the transmission of HAIs-related bacteria, making their hygiene practices essential in preventing infections (13). Moreover, the healthcare environment and medical devices can represent an important reservoir of pathogens. Indeed, contaminated surfaces, instruments, and devices can harbor microorganisms, facilitating thus their persistence and potential transmission to patients, with different materials influencing the adhesion of distinct pathogen classes (14, 15). Eventually, in addition to the characteristics of the pathogens themselves, various environmental factors, including the overall ecosystem of the healthcare setting, and the hygienic conditions of patients and surrounding environment, can significantly impact on the spread of HAIs. In particular, both high temperatures and poor sanitation and inadequate cleaning protocols seem to create an environment conducive to the proliferation of bacteria, thereby increasing the risk of infections (16).

This complexity highlights the need for a more comprehensive approach to infection control. It is estimated that up to 50% of HAIs could be prevented if proper infection prevention and control measures were consistently applied in healthcare settings (17). Among the multidisciplinary interventions implemented to prevent and manage HAIs, strict hygiene protocols have proven to be particularly effective (18–20). However, even nowadays, proper hand washing is followed in less than 40% of cases, even in healthcare units with critically ill patients (21). Therefore, effective prevention strategies must extend beyond hygiene and involve a multidisciplinary collaboration between HCWs, microbiologists, engineers, patients, and visitors, but also a deeper understanding of the factors influencing infection susceptibility. Indeed, only by having a complete understanding of all the players involved and the dynamic interaction between them can it be possible to design targeted preventive strategies that effectively reduce the burden of HAIs and their associated costs (22).

This research involved the administration of a survey to students of the University of Padua, with the aim of exploring awareness, behaviors, and potential risk factors related to the spread of HAIs. This study aims to understand the knowledge that university students, future professionals in different fields, have about HAIs, their transmission, and their awareness of the role they play in preventing them. The aim was not only to identify any gaps in these areas, but also to outline possible targeted training interventions based on the results collected. These insights are essential to design educational programs aimed at improving awareness and prevention of HAIs, as they help identify areas where increased knowledge and targeted interventions are needed, supporting the broader goal of promoting public health. The data collected, based on quantitative and qualitative assessments, constitute a starting point for future studies that intend to deepen and address the problem of HAIs in a more targeted way.

## 2 Materials and methods

### 2.1 Study design and setting

A survey was developed and addressed to the students of the University of Padua enrolled in bachelor’s, master’s, or single-cycle master’s degree courses across different disciplinary areas (i.e., medical, healthcare, biological, scientific, legal, and humanistic). The survey aimed at assessing their level of awareness, opinions, and knowledge on HAIs and the risk of microbial transmission. University students were chosen because they represent a particularly receptive population group, notably malleable with respect to preventive practices and behaviors, including the context of the prevention of HAIs.

### 2.2 Survey development and validation

Prior to large-scale distribution, the survey was administered to a pilot group of 20 students from various degree programs at the University of Padua, for validation. Participants completed the full survey and provided feedback on any unclear or problematic items. Specifically, these 20 students were representatives of the degree courses involved. Therefore, they represented different disciplinary areas, including both healthcare-related and non-healthcare fields, as well as different years of study. This choice was made to ensure that the pilot group would reflect the diversity of the target population. The feedback collected focused on relevance, clarity, and comprehensibility of the survey. Based on this feedback, minor adjustments were made to improve wording and ensure clarity.

### 2.3 Participants, recruitment, and data collection

As the pilot indicated that all items were relevant and, after obtaining authorization from the Presidents of selected degree courses from different disciplinary areas, the survey was distributed via institutional email to a broader student population at the University of Padua.

The survey was administered online using REDCap (Research Electronic Data Capture) tools hosted by the University of Padua from March 2024 to September 2024, reaching approximately 7,000 students (23, 24). Two reminder emails were sent to further encourage participation. The survey was closed 15 days after no additional responses were received. The study involved adult students from the University of Padua and did not collect any clinical or personal health data. Participation was entirely voluntary, and all participants were competent adults. Prior to starting the survey, participants were provided with information regarding the purpose of the study, the anonymity of their responses, and the use of collected data for research purposes. By proceeding to complete the survey, participants implicitly provided their consent to participate and to the processing of their responses.

The survey consisted of 23 questions, divided into four distinct sections, each with specific objectives. Whereas most of the questions were closed-ended, a few included open-ended responses, for specification of pre-defined categories such as “Other occupation,” or “Other chronic illness.” These open-ended questions did not generate narrative responses but rather served to provide additional categorical options for respondents when none of the listed choices applied. The original version of the survey was in Italian, the English translation can be found in the Supplementary material - Data Sheet 1.

The first section was mostly informative, it collected demographic and general information about the participants, such as age, gender, country of origin, and occupation. Additionally, details about students’ educational background and the degree course they were enrolled in were also collected in this section. In the second section, questions explored whether participants or their relatives or loved ones had ever had direct or indirect experience with HAIs, long hospitalizations, or chronic diseases, with reference to events that could have occurred at any point in their lifetime, as no specific recall window was set. This section aims to identify potential situations that could influence their perception of the risk and prevention of HAIs. The third section was designed to assess knowledge and awareness of participants regarding HAIs and related preventive measures, with a particular focus on how the Covid-19 pandemic may have impacted their understanding of the topic. Finally, in the last section, the active engagement of participants in informative campaigns on HAIs and the awareness of their role as visitors in preventing HAIs were evaluated.

### 2.4 Statistical analysis

Survey responses were collected and managed using REDCap and analysed using R (version 4.3.2, Rstudio 2023.10.31 ucrt) (25). Data controls were performed to handle any missing or incorrect values and ensure quality and reliability of the analysed data. In particular, data quality checks were performed to correct any incorrect or inconsistent entries whereas missing responses were treated as such and no imputation was performed, given that the survey items primarily reflected personal experiences or opinions. Analyses were conducted on a question-by-question basis, including only participants who provided a response to the specific item. This approach ensured that each analysis used all available data without introducing assumptions about missing values. The questions of the survey were analysed individually or in association with each other to look for any significant patterns or correlations. In particular, the responses were grouped based on the disciplinary areas or the year of course, to analyse the obtained results. This analysis made it possible to highlight any significant differences in the knowledge of HAIs in relation to the educational background or other personal information. In cases where the survey asked for “other” specifications, these responses were appropriately handled and recategorized. Finally, multivariate statistical analyses were conducted to obtain deeper insights into the interactions between variables and the factors that may influence the knowledge of HAIs. This approach also considered the combined effect of multiple variables, leading to more robust conclusions regarding the impact of different variables on knowledge and awareness in the field of HAIs.

Firstly, a descriptive analysis was performed to explore the characteristics of the sample and the distribution of their responses. This analysis resulted in the creation of frequency or percentage tables and graphs. After the descriptive analysis, univariate analyses were performed for each dependent variable against a set of predefined independent variables, allowing to verify if factors such as direct/indirect hospitalization experiences or relation with chronic diseases may influence the awareness of the HAIs. For each type of dependent variable, appropriate statistical tests were applied to examine the correlations and determine significant associations. When the dependent variable was binary, its relationship with categorical independent variables was assessed using the Chi-square test, unless any expected frequencies were below 5, in which case the Fisher’s exact test was applied. For continuous independent variables, differences between the two groups defined by the binary dependent variable were evaluated using the t-test if the data followed a normal distribution; or the Mann–Whitney U test as a non-parametric alternative. For categorical dependent variables, association with categorical independent variables was assessed using the Chi-square test. If any expected frequencies were below 5, Fisher’s exact test was used instead. Associations with continuous independent variables were tested using one-way ANOVA or Kruskal-Wallis test, depending on whether the data for the independent variable was normally distributed. For continuous dependent variables, parametric tests such as the t-test (for two groups) and ANOVA (for more than two groups) were used to assess differences across categories, with non-parametric alternatives (Mann-Whitney U test or Kruskal-Wallis test). In cases where computational challenges arose due to table size or complexity, a Monte Carlo simulation with 2,000 iterations was used to estimate the *p*-value of univariate analyses.

In the multivariate analysis, more complex models were employed to investigate the combined effects of multiple independent variables on each dependent variable. For binary dependent variables, logistic regression models were fitted to explore associations. A stepwise elimination procedure was applied to remove non-significant variables, ensuring that only the most relevant factors were included in the final model. Variables were considered significant if the *p*-value was below 0.05. For each independent variable in the final model, Odds Ratios (ORs) were calculated to quantify the strength of the association with the dependent variable and 95% Confidence Interval (95% CI) were provided to assess the precision and reliability of the estimates.

For categorical dependent variables with more than two categories, a multinomial logistic regression was applied. A similar stepwise approach was applied to refine the model by removing variables that were not significant, and ORs with corresponding 95% CI were reported for each predictor. For continuous dependent variables, linear regression models were applied, with stepwise elimination retaining only significant predictor. The final model coefficients were interpreted to understand both the magnitude and direction of the relationships. The OR and 95% CI were calculated to assess the precision of the estimates.

All regression analyses were conducted in R (version 4.3.2, RStudio 2023.10.32 ucrt) using functions from the stats, nnet, and car packages. The detailed list of the statistically significant results of all models is reported in Supplementary material - Table A, which includes for each analysis the reference question, the variables considered, the compared and reference categories, standard errors, *p*-values, ORs with their 95% CI, and a brief interpretation. Standard diagnostic tests were performed to check for potential issues such as multicollinearity, linearity, and independence of errors. Variance Inflation Factors (VIFs) were calculated to check for multicollinearity, adopting the conventional threshold of 10 as an indicator of problematic collinearity. In all models, VIF values remained below this threshold. The highest values indicated at most moderate, but still acceptable, levels of collinearity, and therefore no variables were excluded on this basis.

The Akaike Information Criterion (AIC) was used to compare different models, with lower AIC values indicating better fit and predictive accuracy. When comparing multiple groups, corrections for multiple testing were applied using Bonferroni correction to control the family-wise error rate. Adjusted *p*-values (p-adj values) below 0.05 were considered significant, ensuring that post-hoc tests remained valid despite the increased risk of Type I error.

## 3 Results

A total of 1,059 students from the University of Padua answered the survey, although not all of them completed every question. Specifically, all 1,059 students answered questions 1 to 7, while 1,004 provided responses to questions 8 to 16. For questions 17 to 23, the number of respondents slightly decreased, ranging between 1,001 and 1,003. All analyses were conducted using the number of respondents available for each question or combination of variables. Considering the total number of students enrolled in the degree courses to whom the email with the link of the survey was sent, it is estimated that the survey reached approximately 7,000 students, resulting in an estimated response rate of 15.13%. The students’ ages range from 18 to 59 years, with a mean age of 23.4 years and a standard deviation (st. dev.) of 5.5 years. The distribution of students based on age groups on quartiles, gender, nationality, and occupation (Question 1–4) is described in the table below (Table 1).

**Table 1:** Descriptive characteristics of the students.

Response categories (Questions 1–4)	N.	Percentage
Age (Question 1)		
Age ≤ 21	448	42.3%
22 < Age ≤ 22	147	13.9%
23 < Age ≤ 24	271	25.6%
24 < Age	193	18.2%
Total	1,059	100%
Gender (Question 2)		
Female	754	71.2%
Male	292	27.6%
No answer	13	1.2%
Total	1,059	100%
Nation (Question 3)		
Italy	1,019	96.2%
Other	40	3.8%
Total	1,059	100%
Occupation (Question 4)		
Only Student	837	79.0%
PhD/Research Student	28	2.6%
Healthcare worker	61	5.8%
Non-healthcare worker	133	12.6%
Total	1,059	100%

Distribution of students, who answered the survey, based on age groups on quartiles, gender, nation, and occupation.

Focusing on the students’ educational backgrounds (Question 5), it emerged that approximately 54% of respondents attended high schools in the scientific area (*N* = 567), 27% in the humanities area (*N* = 287), 13% in the economic/technological area (*N* = 140), and only 4 and 2% in the professional educational area (*N* = 46) and healthcare area (*N* = 19), respectively.

The results of the distribution of responses by type of degree (Question 6.1), area of study (Question 6.2), and year of study (Question 6.3) are shown in Table 2. These results provided interesting insights into the characteristics of the survey participants, reflecting both their affiliation with different disciplinary areas and how these categories were distributed according to the type of degree and the year of study. When reporting the results obtained, we will refer to students enrolled in the degree course in Medicine and Surgery and the degree course in Dentistry as “Medical area” students, while the term “Healthcare area” will refer to students enrolled in degree courses for non-medical healthcare professions. In all models, if the study area was considered, the Healthcare area is used as the reference category, in other cases the opposite scenario described was used (example: Having a loved one with a chronic disease was compared with not having a loved one with a chronic disease). Among those enrolled in three-year degree courses, the first year was dominated by students from the healthcare area, followed by those from the legal and humanities area and medical area. In master’s courses, the healthcare area remains prevalent, but with an increased presence of the scientific area in the second year. In single-cycle master’s courses, the medical area showed a constant predominance in all years of study.

**Table 2:** Frequency distributions of students according to type of degree (Question 6.1), area of study (Question 6.2), and year of course (Question 6.3).

				Area of study (Question 6.2)				Total
				Healthcare area	Legal and Humanities area	Medical area	Scientific area	
Type of Degree (Question 6.1)	Bachelor’s Degree	Year of Course (Question 6.3)	1	108 (10.2%)	46 (4.3%)	20 (1.9%)	1 (0.1%)	**175 (16.5%)**
			2	50 (4.7%)	12 (1.1%)	12 (1.1%)	3 (0.3%)	**77 (7.2%)**
			3	53 (5.0%)	10 (0.9%)	8 (0.8%)	1 (0.1%)	**72 (6.8%)**
			**Total**	**211 (19.9%)**	**68 (6.3%)**	**40 (3.8%)**	**5 (0.5%)**	**324 (30.5%)**
	Master’s Degree		1	41 (3.9%)	20 (1.9%)	4 (0.4%)	0 (0.0%)	**65 (6.2%)**
			2	28 (2.6%)	5 (0.5%)	4 (0.4%)	9 (0.8%)	**46 (4.3%)**
			**Total**	**69 (6.5%)**	**25 (2.4%)**	**8 (0.8%)**	**9 (0.8%)**	**111 (10.5%)**
	Single-Cycle Master’s Degree		1	4 (0.4%)	7 (0.7%)	99 (9.3%)	1 (0.1%)	**111 (10.5%)**
			2	9 (0.8%)	6 (0.6%)	103 (9.7%)	0 (0.0%)	**118 (11.1%)**
			3	5 (0.5%)	12 (1.1%)	119 (11.2%)	0 (0.0%)	**136 (12.8%)**
			4	6 (0.6%)	20 (1.9%)	62 (5.9%)	1 (0.1%)	**89 (8.5%)**
			5	17 (1.6%)	7 (0.7%)	103 (9.7%)	1 (0.1%)	**128 (12.1%)**
			6	8 (0.8%)	2 (0.2%)	32 (3.0%)	0 (0.0%)	**42 (4.0%)**
			**Total**	**49 (4.7%)**	**54 (5.2%)**	**518 (48.8%)**	**3 (0.3%)**	**624 (59.0%)**

This table reports the number and the percentage of respondents grouped by degree type (bachelor’s, master’s, and single-cycle master’s degrees) and by year of study (from 1 to 6, where applicable), in relation to each academic area considered (healthcare, legal and humanities, medical, and scientific areas).

The analysis of the years of enrolment (Question 6.4) revealed that, for the majority of students, the academic career was proceeding regularly, indicating that a significant portion of the sample is on track to complete their courses as expected. Additionally, based on the responses on Question 6.5.1, 95 students (9.0%) already hold a degree in the healthcare area, 40 (3.8%) in legal and humanities area, 27 (2.5%) in the scientific area, and 13 (1.2%) in the medical area.

Regarding Question 7, which asked whether students were aware of what HAIs were before reading the provided definition, a total of 984 positive answers (92.9%) and 75 negative answers (7.1%) were recorded. The distribution of these responses, divided by study area (Question 6.2) and year of study (Question 6.3), is presented in Table 3. Although subsequent analyses were performed to evaluate the effect of different independent variables on the knowledge of what HAIs are, an initial examination assessed the influence of factors such as area of study and academic year, given that academic training can significantly shape awareness of health issues such as HAIs (26–28). In particular, to explore whether there was a correlation between students’ declared knowledge of HAIs (yes/no) and their study area, a Fisher’s exact test was performed on the contingency table that compared the responses across the four study areas: healthcare area, legal and humanities area, medical area, and scientific area. The test revealed a highly significant difference between the study areas (*p*-value = 3.08 × 10–12). Subsequently, to further investigate the differences between these groups, a post-hoc test with Bonferroni correction was performed. The results showed that students from the legal and humanities areas significantly different from those in the healthcare area (*p*-adj value = 1.58×10-9) and those in the medical area (*p*-adj value = 2.97×10-11). No other significant differences emerged considering the study areas.

**Table 3:** Frequency distributions of students based on their knowledge of the definition of HAIs (Question 7), area of study (Question 6.2), and year of course (Question 6.3).

				Area of study (Question 6.2)				Total
				Healthcare area	Legal and Humanities area	Medical area	Scientific area	
HAIs knowledge (Question 7)	NO	Year of Course (Question 6.3)	1	7 (0.7%)	23 (2.1%)	20 (1.9%)	0 (0.0%)	**50 (4.7%)**
			2	2 (0.2%)	2 (0.2%)	2 (0.2%)	0 (0.0%)	**6 (0.6%)**
			3	2 (0.2%)	5 (0.5%)	2 (0.2%)	0 (0.0%)	**9 (0.9%)**
			4	0 (0.0%)	3 (0.3%)	0 (0.0%)	0 (0.0%)	**3 (0.3%)**
			5	2 (0.2%)	2 (0.2%)	0 (0.0%)	0 (0.0%)	**4 (0.4%)**
			6	1 (0.1%)	1 (0.1%)	1 (0.1%)	0 (0.0%)	**3 (0.3%)**
			**Total**	**14 (1.4%)**	**36 (3.4%)**	**25 (2.4%)**	**0 (0.0%)**	**75 (7.2%)**
	YES		1	146 (13.8%)	50 (4.7%)	103 (9.7%)	2 (0.2%)	**301 (28.4%)**
			2	85 (8.0%)	21 (2%)	117 (11%)	12 (1.1%)	**235 (22.1%)**
			3	56 (5.3%)	17 (1.6%)	125 (11.8%)	1 (0.1%)	**199 (18.8%)**
			4	6 (0.6%)	17 (1.6%)	62 (5.8%)	1 (0.1%)	**86 (8.1%)**
			5	15 (1.4%)	5 (0.5%)	103 (9.7%)	1 (0.1%)	**124 (11.7%)**
			6	7 (0.7%)	1 (0.1%)	31 (2.9%)	0 (0.0%)	**39 (3.7%)**
			**Total**	**315 (29.8%)**	**111 (10.5%)**	**541 (50.9%)**	**17 (1.6%)**	**984 (92.9%)**

Respondents are grouped by whether or not they reported knowing what HAIs are, and further classified by year of course and the academic area of study (healthcare, legal and humanities, medical, and scientific areas).

A structured approach was then applied to study the relationships among knowledge of HAIs and variables associated with students and their relatives/loved ones.

**Figure 1:**
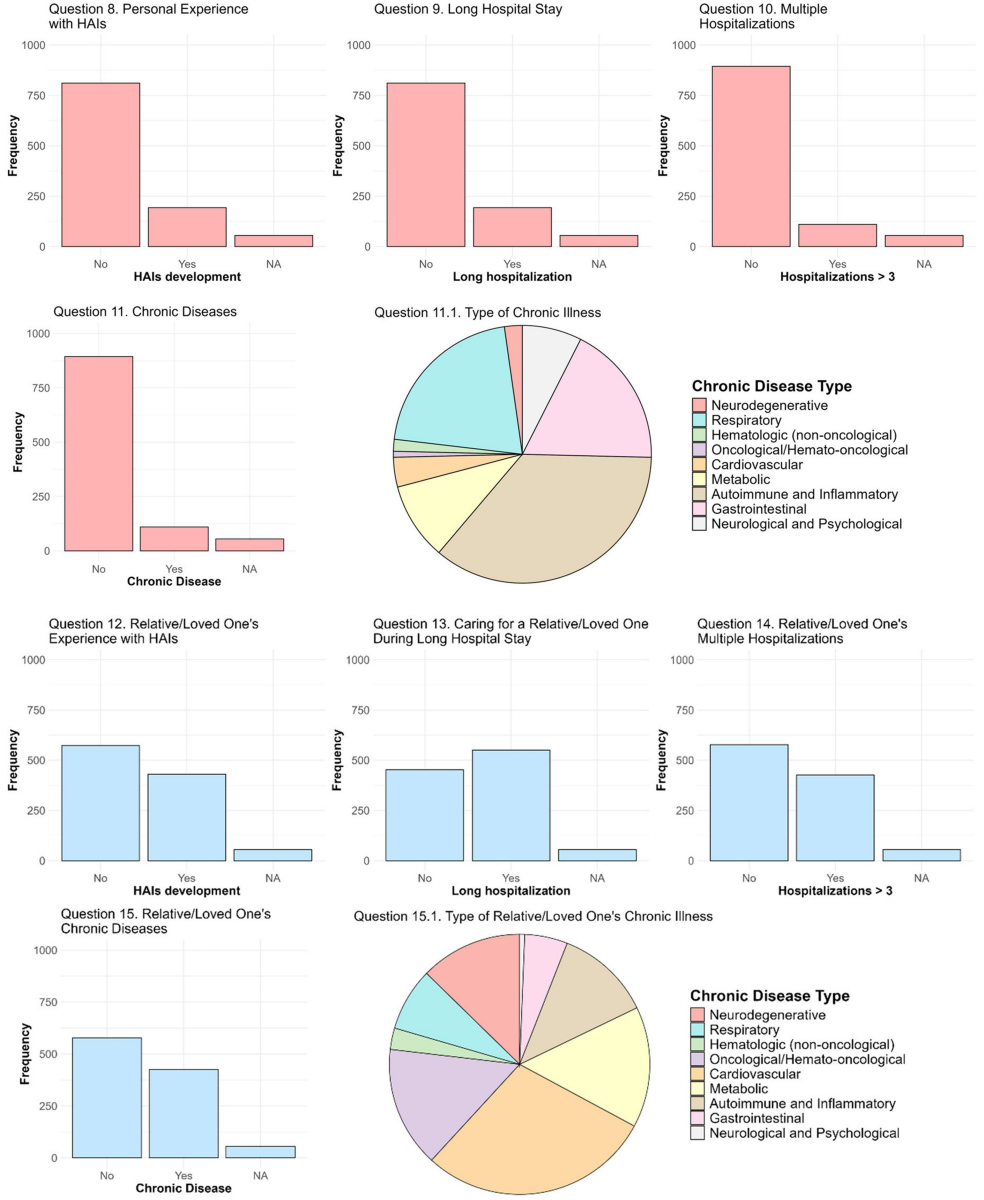
Medical history of students and their relatives/loved ones. The responses to Questions 8, 9, 10, and 11 are shown in pink bar charts, addressing the development of HAIs in students, the occurrence of a long hospitalization, the number of prolonged hospitalizations, and the presence of chronic diseases, respectively. The pie chart (Question 11.1) depicts the distribution of the types of chronic diseases reported by students. The responses to Questions 12, 13, 14, and 15 are shown in light-blue bar charts, addressing the following aspects: the development of HAIs in a student’s relative/loved one, the need to take care of a students’ relative/loved one during one long hospitalizations, the number of prolonged hospitalizations in a student’s relative/loved one, and the presence of chronic diseases in a student’s relative/loved one, respectively. The pie chart (Question 15.1) illustrates the distribution of the types of chronic diseases reported for the students’ relatives/loved ones.

The analysis began with a univariate examination of the responses to Question 7 and other independent variables. These variables included not only factors related to the students’ academic background but also aspects related to their medical histories and those of their relatives/loved ones (Figure 1). The analysis of the final model in terms of OR and 95% CI led to the following results:

- students in the legal and humanities area had a significantly lower probability of knowing what HAIs are, compared to the students in the healthcare area with an OR of 0.12 (95% CI 0.06;0.2);
- students were significantly more likely to be aware of HAIs as the years of their course progress, with an OR of 1.53 (95% CI 1.26;1.90);
- students with a relative/loved one who has at least one chronic disease or who has taken care of a relative/loved one during a long hospital stay (> 5 days) had an increased probability to know the meaning of HAIs, with an OR of 2.17 (95% CI 1.20;4.12) and 2.01 (95% CI 1.14; 3.69), respectively.

The responses to the questions on students’ medical history and on that of their relatives/loved ones are reported in Figure 1. Notably, the data revealed significant differences between students and their relatives/loved ones regarding HAIs (*p*-value = 0.0035) and the presence of chronic diseases (*p*-value = 8.11×10^−6^), with a higher incidence observed in relatives/loved ones. Regarding the development of an HAIs in the students (Question 8, *N* = 1,004), almost all students (97.7%, *N* = 981) declared that they had never contracted HAIs, whereas 2.3% (*N* = 23) indicated that they had. Similarly, in Question 9 (*N* = 1,004), most students denied having had a long hospitalization of more than 5 days (80.8%, *N* = 811), even though 19.2% (*N* = 193) reported experiencing this. For the number of prolonged hospitalizations (Question 10, *N* = 1,004), 94.6% (*N* = 950) of students stated they had never experienced more than three hospitalizations lasting at least 5 days, with only 5.4% (*N* = 54) reporting otherwise. Regarding chronic diseases (Question 11, *N* = 1,004), it emerged that 89% of students (*N* = 894) did not suffer from any chronic disease, whereas 11% (*N* = 110) indicated they had at least one chronic condition. The most prevalent conditions (Question 11.1) were autoimmune diseases and other inflammatory disorders (48 cases), followed by respiratory (28 cases), gastrointestinal (24 cases), and metabolic diseases (13 cases). Less common conditions included neurological and psychological diseases (10 cases), cardiovascular (5 cases), neurodegenerative (3 cases), hematologic (non-oncological) (2 cases), and other oncological and hemato-oncological diseases (1 case).

With regard to HAIs (Question 12, *N* = 1,004), 24.2% of students (*N* = 243) reported that a relative/loved one has been affected, compared to 75.8% (*N* = 761) who reported no cases. In Question 13, 42.8% of students (430 over 1,004) reported having to care for a relative/loved one during a long hospital stay. Additionally, 54.9% of respondents (551 over 1,004) noted that a relative/loved one had been hospitalized more than three times for periods longer than 5 days (Question 14). Finally, regarding chronic diseases (Question 15), 42.4% of students (426 over 1,004) reported that a relative/loved one suffers from at least one chronic disease. Regarding the responses to Question 15.1, cardiovascular diseases were the most common among relatives (185 cases), followed by oncological and haemato-oncological (96 cases), metabolic (96 cases), and neurodegenerative diseases (81 cases). Autoimmune diseases and other inflammatory disorders (76 cases) and respiratory diseases (50 cases) are also relevant, whereas gastrointestinal diseases (34 cases), hematologic (non-oncological) diseases (17 cases), and neurological and psychological diseases (4 cases) were less frequent.

Analysing the number of reported chronic diseases, the majority of affected students reported having a single chronic disease, whereas the number of chronic diseases reported for their relatives/loved ones was consistently higher, with some students reporting up to five chronic conditions for their relatives/loved ones.

**Figure 2:**
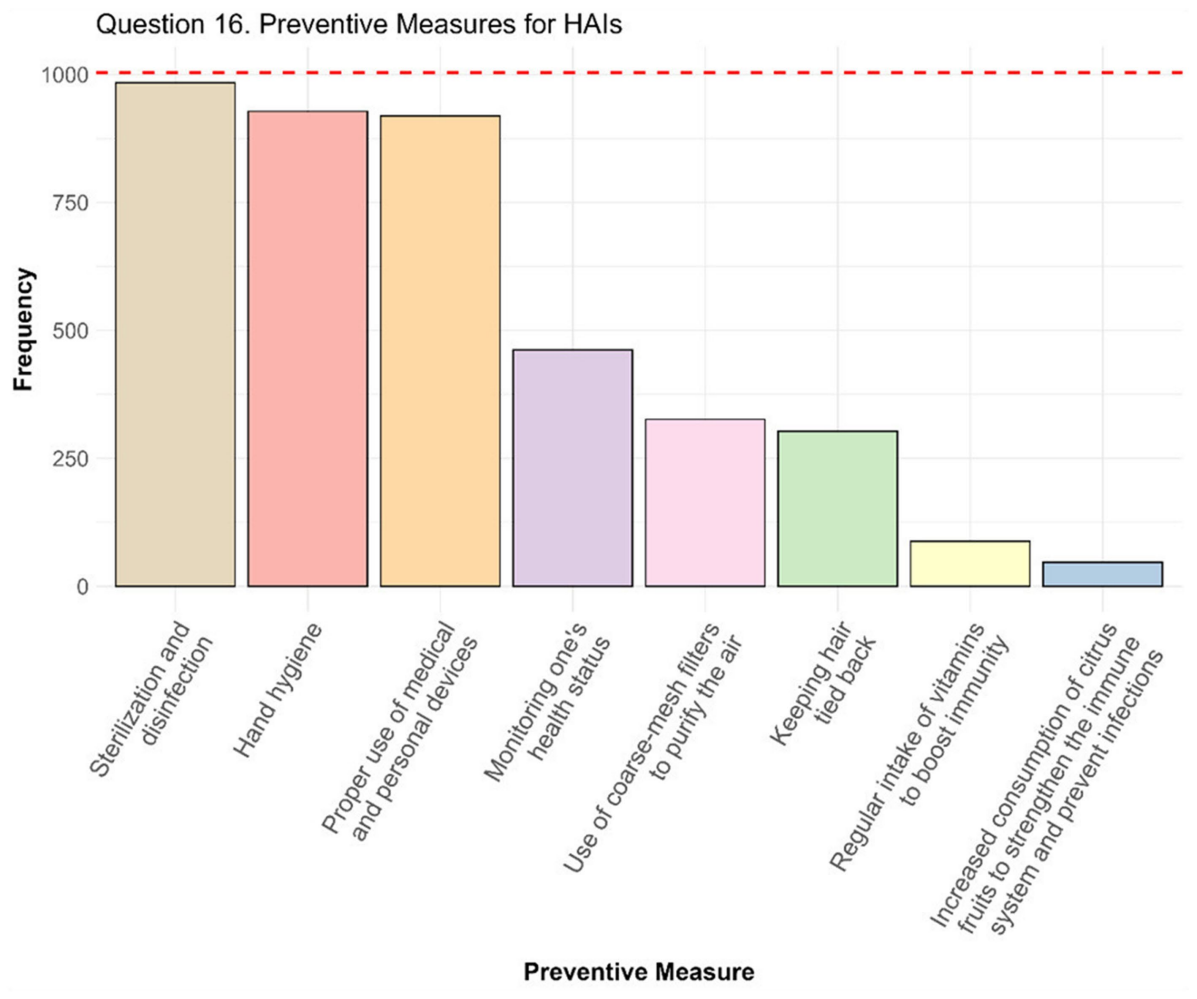
Preventive measures for HAIs. In the bar chart the number of responses obtained for each preventive measure are represented. The red dot line represents the total number of students who answered the question, thus the maximum number of individuals who could have selected each option.

The responses to Question 16 (*N* = 1,004) provided valuable insights into the preventive measures considered by students to be most effective in reducing the incidence of HAIs (Figure 2). Each student had the possibility to select multiple answers, providing a detailed profile of perceived priorities. The three most frequently selected options – sterilization and disinfection, hand hygiene, and proper use of medical and personal devices – were selected by 984 (98.0%), 928 (92.4%), and 919 (91.5%) students over a total of 1,004, respectively. For further analysis, these three options were considered the most relevant ones, as they are widely recognized as fundamental for the prevention of HAIs in the healthcare context (19, 29, 30). Other, although less frequent, answers were indicated as preventive measures by a considerable number of students, including monitoring health status (462 students, 46.0%), using coarse-mesh filters to purify the air (326 students, 23.5%), and keeping hair tied back (303 students, 30.2%).

The distribution of the number of options selected highlights variability in how students perceive the comprehensiveness of prevention. In particular, among the students who responded, 9 students (0.9%) selected only one preventive measure, and 46 students (4.6%) indicated two. Most students chose three or four options: 304 students (30.3%) selected three measures, and 333 students (33.1%) selected four. Another considerable number of participants (213 students, 21.2%) chose five preventive measures, whereas a minority, 64 students (6.4%), selected six options. Finally, 17 students (1.7%) chose seven options, and 18 students (1.8%) indicated all eight available preventive measures as important. A Fisher’s exact test was employed to assess the association between study area/year of study and the selection of only the three primary preventive measures and the results revealed no significant differences in either cases (*p*-value was 0.11 for the study area and 0.95 for the year of study).

**Figure 3:**
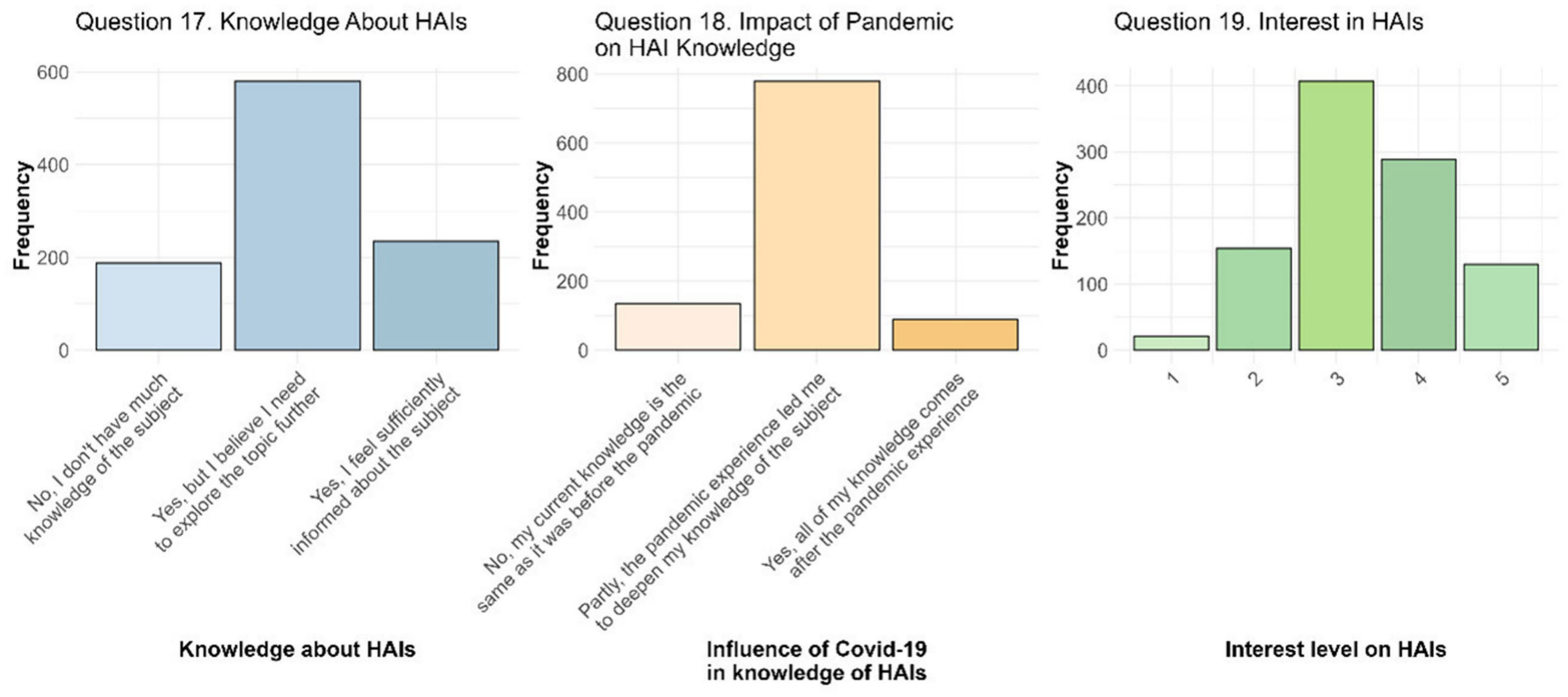
Awareness and knowledge of HAIs. The image presents responses to Question 17, which explores students’ personal perception of their knowledge about HAIs; Question 18, which highlights the impact of the COVID-19 pandemic on their understanding of HAIs; and Question 19, which reflects what students think is the current interest on HAIs.

Questions 17, 18, and 19 examined students’ self-perceived knowledge of HAIs, the impact of the Covid-19 on this knowledge, and perceptions of public interest in HAIs (Figure 3).

Regarding feeling sufficiently informed about HAIs and their management (Question 17, *N* = 1,003), the majority of students (*N* = 580, 57.8%) stated that they were familiar with the topic, although they felt they need to learn more about it. In contrast, 235 students (23.4%) felt adequately informed, whereas 188 students (18.8%) admitted to having limited knowledge of the topic. These responses were then analysed in relation to the different personal and medical information on students and their relatives/loved ones. After the structured approach previous proposed – composed by univariate analysis, significant variable identification, multivariate model design and evaluation – ORs and their 95% CI were calculated. Having as reference the first answer “No, I do not have much knowledge of the subject,” students in the legal and humanities area had a significant lower probability to give the other two responses with OR of 0.10 (95% CI 0.06; 0.17) for “Yes, but I believe I need to explore the topic further” (second answer) and OR of 0.03 (95% CI 0.01; 0.08) for “Yes, I feel sufficiently informed about the subject” (third answer), compared to the students in healthcare area. Although still lower compared to students in the healthcare area, both medical and scientific area students showed significant results with OR of 0.60 (95% CI 0.38; 0.96) and OR of 0.52 (95% CI 0.31; 0.88) for medical area and OR of 0.15 (95% CI 0.04; 0.54) and OR of 0.11 (95% CI 0.02; 0.52) for scientific area, respectively for second and third answer. Moreover, the probability of feeling increasingly informed rose with progression in academic year l with OR of 1.36 (95% CI 1.12; 1.67). Students who had cared for a relative/loved one during a long hospitalization were also more likely to believe that they needed to explore the topic further (OR = 1.53, 95% CI 1.05; 2.22).

The Covid-19 pandemic has undoubtedly had a significant influence on the perception of HAIs for students as highlighted by the responses to Question 18 (*N* = 1,002). Specifically, the 86.6% of respondents stated that the pandemic experience contributed to broadening their knowledge on the topic (779 students, 77.7%), or that all their knowledge on HAIs was acquired during and after the pandemic began (89 students, 8.9%). Only 134 students (13.4%) reported that the pandemic had no impact on their knowledge. The broad impact of the Covid-19 pandemic on knowledge of HAIs was found to be significantly influenced by the area of study of students. In all cases, the healthcare area was used as the reference category. Therefore, students in legal and humanities area, the scientific area, and the medical area showed a trend toward a lower probability of giving the second answer compared to students belonging to healthcare area, with ORs of 0.27 (95% CI 0.15; 0.49) for legal and humanities area, 0.28 (95% CI 0.08; 0.98) for scientific area, and 0.60 (95% CI 0.37; 0.98) for medical area. Considering the third answer only legal and humanities area resulted still significant comparing to healthcare area with an OR of 0.14 (95% CI 0.05; 0.42).

The responses to Question 19 (*N* = 1,001) allow us to understand what students believe to be the current interest in HAIs, measured on a scale from 1 to 5. The distribution of the responses is centered on medium-high values, with a mean ± st. dev. of 3.35 ± 0.96. Following the structured approach of univariate analyses, identification of statistically significant variables, and linear regression model definition with stepwise selection, several predictors emerged as significant. In particular, students with more than 24 years believed there is a higher level of interest in HAIs compared to those with less than 21 years (OR = 1.26, 95% CI 1.07; 1.49), similarly to those with a relative/loved one with at least one chronic disease (OR = 1.16, 95% CI 1.03; 1.30). Differently, students in the legal and humanities area (OR = 0.57, 95% CI 0.47; 0.69) and those in the scientific area (OR = 0.54, 95% CI 0.33; 0.87) believe there is a significantly lower interest on HAIs, rather than students in the healthcare area (reference group).

**Figure 4:**
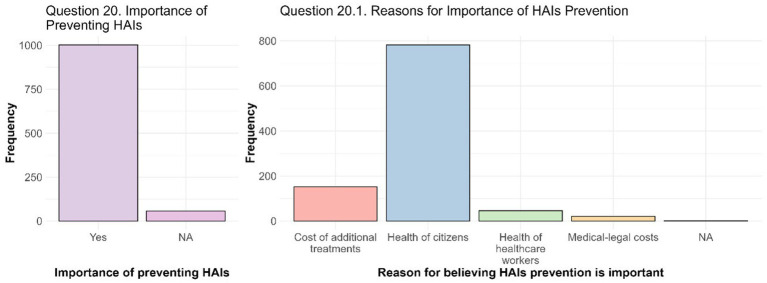
HAIs prevention and its importance. The importance of preventing HAIs is depicted by an only selected answer to Question 20. Considering the reason for believing HAIs prevention important (Question 20.1), health of citizens represent the most considered reason.

For the analysis of Questions 20 and 20.1, the main goal was to assess the students’ perception of the importance of preventing HAIs and their reasons for prioritizing prevention (Figure 4). Question 20 asked whether students considered the prevention of HAIs to be important. A total of 1,002 students out of 1,059 responded to this question, and all of them answered “Yes,” demonstrating a unanimous consensus on the importance of addressing this issue. Question 20.1 (*N* = 1,001) then asked students to explain their reasoning for considering HAIs prevention important. Four options, linked to the effects of these infections on different aspects of the health system and society, were provided, and the students who answered “Yes” to Question 20 were asked to choose the most relevant motivations for them. The responses revealed a clear priority given to the health of citizens in general, with 782 responses (78.0% of the total) identifying this aspect as the main reason for preventing HAIs. Economic motivations follow, with 152 responses (15.2%) highlighting the weight of HAIs on the costs sustained by the national health system related to the additional treatments required. Less attention was given to the protection of the health of HCWs (46 responses, 4.6%) and to the costs associated with medical-legal disputes (21 responses, 2.1%). The structured approach allowed to reveal that students that already graduated give more importance to the health of citizens rather than to the cost of additional treatments (OR = 2.21, 95% CI 1.29; 3.78). With the increasing of the year of course, there is a significantly reduced likelihood of preferring the response associated with the health of HCWs rather than those associated with the cost of additional care with OR = 0.58 (95% CI 0.41; 0.82), whereas students who have a relative/loved one with at least one chronic disease are more likely to prioritize the health of HCWs compared to the costs (OR = 2.18, 95% CI 1.10; 4.31).

Although most students recognize the need to deepen their knowledge on HAIs, it seems that they are largely unaware of information campaigns on the topic (Question 21, *N* = 1,003). In fact, only 194 students (19.3% of the total) stated of being aware of any training events of this kind. However, of these on Question 22, only 18 students confirmed they had taken part. Interestingly, another 8 students stated that they had participated in information campaigns on HAIs, even though they indicated they were not currently aware of any ongoing training events on the topic. The analysis which led to the production of a multivariate logistic regression model on the associations with the knowledge of information campaigns highlighted different significant associations. Students of the fourth quartile of age are about twice as likely to be aware of the campaigns as the youngest quartile (OR = 2.18, 95% CI 1.34; 3.53). Students without other occupations (OR = 0.24, 95% CI 0.13; 0.45), PhD/research students (OR = 0.20, 95% CI 0.05; 0.63), and non-HCWs (OR = 0.35, 95% CI 0.17; 0.68) revealed a lower probability to be aware of the campaigns compared to the HCWs, as well as, students in legal and humanities area compared to those of healthcare area (OR = 0.30, 95% CI 0.13; 0.61). Interesting to notice that having a relative/loved one with a higher number of chronic diseases (OR = 1.24, 95% CI 1.03; 1.48) increase the probability to know existing information campaigns at each additional chronic disease. Moreover, although with a borderline significance (*p*-value = 0.05), having experienced HAIs increase the probability to know information campaigns (OR = 2.55, 95% CI 0.97; 6.53). On the other hand, considering the participation to information campaigns (Question 22), it seems that students who already have a degree are less likely to participate (OR = 0.25, 95% CI 0.10; 0.64).

**Figure 5:**
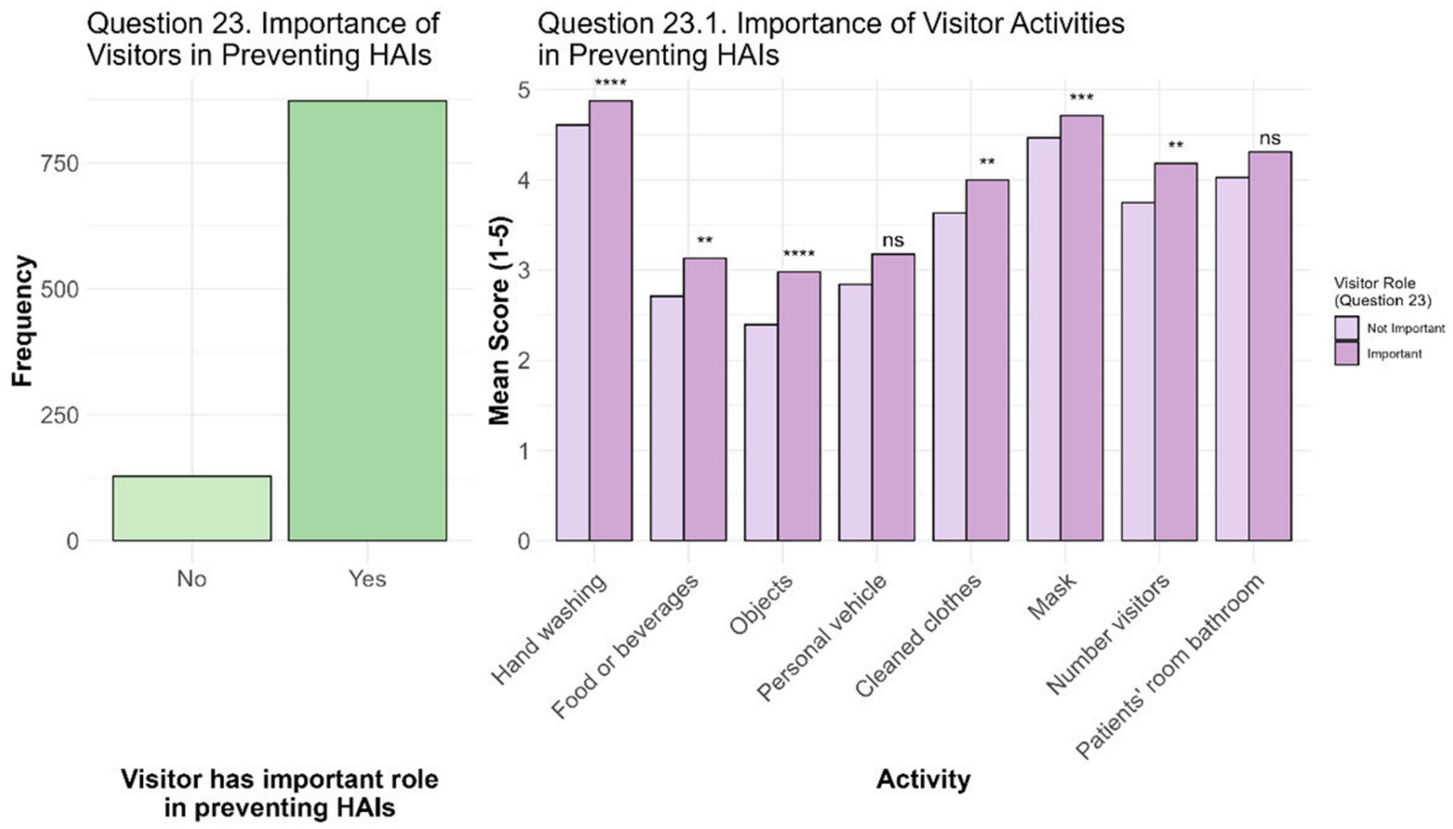
Importance of visitor in preventing HAIs. The bar chart on the left, refers to Question 23, displayed the number of students who perceives the role of visitors as not important (No) or important (Yes) in the context of HAIs prevention. Instead, on the right (Question 23.1), the different mean scores are shown for each activities considering the student differentiation based on the answer to Question 23. For each activity comparison of non-parametric data were performed with Mann–Whitney U tests and Bonferroni adjustment: * *p*-adj value < 0.05, ** *p*-adj value < 0.01, *** *p*-adj value < 0.001, and **** *p*-adj value < 0.0001.

The last question (*N* = 1,001) consisted of two parts: Question 23, which explored students’ perceptions of the role of hospital visitors in the prevention of HAIs; and Question 23.1, where students were asked to rate from 1 to 5 the importance of specific visitors’ activities in the prevention of HAIs (Figure 5). The vast majority of students (87.2%, *N* = 873) believe that visitors play an important role in this context, whereas a small minority (12.8%, *N* = 128) does not recognize their importance. However, when the logistic regression model was designed and adjusted, significant differences emerged. All the students who work as HCWs have a higher probability of giving importance to the visitor in the context of HAIs prevention. In particular, compared to HCWs, students without work have an OR of 0.11 (95% CI 0.01; 0.51), students who are also PhD students/researchers have an OR of 0.09 (95% CI 0.01; 0.69), and students who work as non-HCWs have an OR of 0.10 (95% CI 0.01; 0.48). Moreover, the probability of attributing a significant role to the visitor in the context of infection prevention is higher in the case in which the students had to take care of a relative/loved one during a long period of hospitalization (> 5 days) with an OR of 1.53 (95% CI 1.04; 2.29). Interestingly, this is the only variable that resulted in being influenced also by the gender of the respondents, since males seem less inclined to consider the role of visitors important in the prevention of HAIs with an OR of 0.49 (95% CI 0.34; 0.73).

Moving to the relevance that students give to the impact of visitor activities on HAIs, mean scores indicate a strong emphasis on hygiene and health measures such as hand washing (mean ± st. dev. = 4.84 ± 0.43), the use of masks as personal protective equipment (mean ± st. dev. = 4.68 ± 0.63), and avoiding the use of the patients’ room bathroom (mean ± st. dev. = 4.27 ± 0.98). Other measures, such as limiting the number of visitors (mean ± st. dev. = 4.13 ± 0.98) and wearing clean clothes (mean ± st. dev. = 3.95 ± 1.01), also received relatively high mean ratings. However, other practices such as avoiding bringing food or drinks (mean ± st. dev. = 3.08 ± 1.15), using private vehicles (mean ± st. dev. = 3.13 ± 1.28) and limiting the transportation of personal objects (mean ± st. dev. = 2.91 ± 1.14), were deemed less relevant by the students. Although the average scores attributed to these activities were generally high, significant differences emerged between the rating of students who considered the visitor’s role unimportant and those who viewed it as fundamental. This was highlighted by the Mann–Whitney U tests and Bonferroni adjustment, which showed that the groups differed significantly for the majority of the activities analysed (*p*-adj values < 0.05). Although the average ratings given to the different activities are quite high, they are still lower among students who do not consider the role of visitors important in preventing HAIs compared to those who recognize the importance of visitors in the healthcare context.

## 4 Discussion

The analysis of the survey results allowed to collect fundamental data on the awareness, knowledge, and behaviors of Padua University students in relation to HAIs. This study offers a preliminary overview of the level of preparation and the demographic and academic characteristics of the students involved, helping to identify possible training gaps and propose targeted strategies to improve the prevention and control of infections in the healthcare sector. The results obtained provide an important basis to identify parameters associated with students’ awareness about HAIs, such as the area of study and academic advancement. Through these data, it is possible to reflect on the educational and organizational implications necessary to promote greater awareness and virtuous behaviors, not only among students of healthcare areas, but also in other academic areas.

The sample of 1,059 students who participated in the survey represents approximately 15.13% of the students reached via email. Although this response rate is moderate, it aligns with other online surveys in academic settings, where participation rates often range between 10 and 30%. For instance, Pedersen et al., using the same data collection platform (REDCap) for a survey on perceptions of HAIs, achieved a response rate of 12.2% (31). Similarly, the trends reported by Muñoz-Leiva et al. support our findings, as they observed that response rates for email surveys have declined significantly over time – from over 50% in the early 1990s to much lower levels today – due to the widespread use of filters and increasing survey fatigue (32). Respondents were drawn from the same degree courses originally targeted, and the gender distribution in our sample (71.2% female) was reasonably consistent with that of the underlying student population (about 67% female). Nevertheless, the absence of detailed information on other characteristics of non-responders, such as age, limits the possibility of fully assessing representativeness. The wide age range of participants (18–59 years) reflects the variety of academic and personal backgrounds among students at the University of Padua. The mean of 23.4 years, with a standard deviation of 5.5, reveals that the majority of the sample is composed of young students. Moreover, considering the demographic distribution of the respondents, the majority are female who have no occupation (Table 1). This is in line with the report of MIUR (Italian Ministry of University and Research) which stated that women consistently represent more than 50% of the Italian university student population (33). The prevalence of students with a scientific educational background (54%, *N* = 567) is consistent with the tradition of Italian universities, where scientific high schools represent a common preparation path for university studies, particularly in STEM (Science, Technology, Engineering and Mathematics) and healthcare areas. These data are also in accordance with research that highlights how the pre-university background significantly influences the choice of academic path and future specialization (34). Similarly, the area of study is consistent with the type of degree in accordance with the Italian university system (Table 2). The high percentage of positive responses (984 out of 1,059, approximately 93%) regarding the knowledge of HAIs (Question 7) indicates a good level of awareness among students, probably due to the growing public and media attention toward HAIs, especially during and after the Covid-19 pandemic (35, 36). Indeed, although not being an HAI in the strict sense, Covid-19 had a significant and transversal impact on education and awareness of infection prevention in general, including HAIs, thus contributing to a greater awareness among students on this issue. This consideration is supported by the responses to Question 18 (Figure 3) where students revealed a significant impact of the pandemic on their knowledge of HAIs: 86.6% of students (N = 868) reported that they had gained greater awareness of the topic due to the pandemic, with only 13.4% that reported that they had not perceived any change in their knowledge. Students in the legal/humanities, scientific, and medical areas were less likely to perceive an increase in their knowledge than students in the healthcare area. This may reflect a lower direct impact of the pandemic in non-healthcare and non-medical areas, where practical experience and exposure to the topic of HAIs may have been more limited, as suggested by different studies (35, 37, 38). However, despite their moderate responses to the second question, students in the science and medical area, similarly to healthcare students, still reported an association between their learning on the topic more directly with the pandemic experience. This may be due to the practical and academic context that encourages them to study and explore deeper this topic. In light of this, knowledge about HAIs seems to be correlated with several factors, including the area of study, the year of course (Table 3), and the medical history of students’ relatives/loved ones. Similarly, although the majority of students (*N* = 580, 57.8%) reported being familiar with the topic of infections and their prevention, while still needing further exploration, the responses to Question 17 (Figure 3) were also found to be related to the area of study, the year of study, and the care of a relative/love one during a long hospitalization. This data, in accordance with literature, indicates an initial awareness of the importance of these infections and that increasing the knowledge on this topic is of paramount importance (27, 39, 40).

The lower knowledge and the reduced likelihood of feeling informed about HAIs among students from the legal and humanities area, compared to those from healthcare area, is justified on the basis of different contents, structure, and purposes of these studies paths. This disparity underscores the varying degrees of exposure to health-related issues within different educational tracks. These findings highlight the importance of integrating elements of health education into university courses not directly connected to medicine or healthcare. Interestingly, previous studies have already observed this trend and have recommended incorporating specialized curricula on HAIs and broader infection prevention topics across diverse disciplines (27, 28, 41–44). This approach could enhance cross-disciplinary awareness, fostering a more collaborative and inclusive strategy for managing infections in both healthcare and community settings. The rationale for broadening this educational scope lies in the role everyone plays in preventing HAIs, as emphasized by different studies, which argue that education on these topics should not be limited to HCWs but should also involve patients, their families, and, more broadly, the entire population (27, 28, 41, 42). This comprehensive involvement is crucial for addressing HAIs as a widespread public health challenge. Surprisingly, also students in medical and scientific areas showed a low probability of feeling informed about HAIs compared to those in the healthcare area. This aligns with other literature studies, which have observed a higher level of knowledge on the topics among healthcare students compared to those of other areas (38, 42, 43, 45). The positive correlation between the years of study and both knowledge and feeling sufficient informed about HAIs was expected, and this was confirmed by our statistical analysis. From HAIs prevention perspective, these data are encouraging, as they suggest that as students’ educational and cultural levels increase over the years, so does their awareness of important issues with high social impact, such as HAIs, and the knowledge about them. Eventually, having cared for a relative/loved one during a long hospitalization proved to be associated with higher awareness of HAIs and also helped to recognize the need to further explore the topic. In addition, although not associated with the feeling of being informed about HAIs, having a relative/loved one with at least one chronic disease, or who has experienced HAIs, was also demonstrated to be a factor associated with increased awareness of the importance of HAIs. These findings suggest that, beyond the cultural level and beyond theoretical knowledge, personal life experiences always play a crucial role (37). Barratt et al. emphasized that patients’ understanding of infection control often arises from their own hospitalization experiences, which shape their awareness and perceptions of healthcare practices (39). Similarly, Mitchell et al. highlighted how the sociocultural context of having experienced with HAIs shapes not only patients’ understanding of HAIs but also their interactions with HCWs (46). Whereas these studies focused on direct patient experiences, it is plausible that indirect exposure, such as caregiving or witnessing a relative/loved one’s experience, may also have a critical role in enhancing knowledge and shaping perceptions of HAIs, as highlighted by this analysis. This is especially true considering that, the overall average incidence of HAIs obtained is approximately 13.25%, these data results to be consistent with the average about 12.4% reported by Miller et al. (40). The answers to Question 16 (Figure 2) provide an in-depth overview of the priorities perceived by students regarding preventive measures against HAIs. The results highlight a good awareness of fundamental measures, such as sterilization and disinfection (984 selections, 98.0%), hand hygiene (928 selections, 92.4%), and the correct use of medical and personal devices (919 selections, 91.5%). The priority given to these practices is in line with what is suggested by scientific literature and international recommendations for the prevention of HAIs, which underline their importance in reducing the risks of infection in healthcare settings (19, 27, 28, 37, 44, 47–49). Furthermore, our results show that the knowledge about these preventive measures does not seem to be influenced by specific factors related to the students themselves. Such consensus could, very likely, be attributed to the broader context of the Covid-19 pandemic, which has contributed to highlight and make more recognized the importance of these practices (29, 30, 35, 36). However, the analysis of the distribution of the number of preventive measures selected highlights a diversity in students’ perceptions regarding their importance. Only a minority (9 students, 0.9%) indicated a single preventive measure, whereas the majority selected three or four options, indicating that many students perceive prevention as a multifactorial strategy. However, some confusion regarding the relevance of some preventive measures is also evident, as demonstrated by responses that include less scientifically relevant options (e.g., citrus fruit consumption or vitamin intake). Overall, these data suggest that, although students have a good understanding of basic strategies, it would be appropriate to implement interventions, also in this sense, aimed at promoting a deeper understanding of the topic (27, 28, 41–44).

When asked about what they thought to be the current interest in HAIs on a scale of 1 to 5, where 1 indicated no interest and 5 maximum interest, students answered with an average of 3.35 ± 0.96 (st. dev.) (Question 19 - Figure 3). Although students think that, currently, there is a moderate interest in the topic, more in-depth analyses revealed that various factors influenced this perception, such as age, area of study, and having a relative/loved one with at least a chronic disease. In particular, the area of study is crucial: students in the legal and humanities area and those in scientific area showed a lower propensity to perceive the general interest in HAIs as relevant compared to their colleagues in the healthcare area (OR = 0.57, 95% CI 0.47; 0.69). On the contrary, direct or indirect personal experiences, such as, in this case, having a relative/loved one affected by at least one chronic disease, seem to strengthen what students think is the interest and awareness toward HAIs (OR = 1.16, 95% CI 1.03; 1.30). Similarly, age has shown a significant impact: students over 24 years of age have a higher propensity to perceive HAIs as relevant (OR = 1.26, 95% CI 1.07; 1.49). This finding suggests that greater maturity, combined with personal experiences accumulated with age, could favor greater attention to health issues such as HAIs. Although this result may seem predictable, the role of age remains controversial in literature. For example, the study by D’Alessandro et al. found that younger students (≤24 years) in the health field show higher levels of knowledge than their older colleagues. The authors hypothesize that this may depend on a greater attendance at lessons by young people or faster and more effective training processes. Despite this observation, the study recognizes the need for further investigation, suggesting that the influence of age could also derive from incorrect learned behavioral patterns (38). On the other hand, other studies, such as that of Wu et al., have highlighted a significantly positive relationship between increasing age and knowledge scores related to HAIs, supporting the idea that experience accumulated over time can increase awareness (35, 37, 50). This divergence in results highlights the complexity of the relationship between age and knowledge of HAIs, and in this case the perceived interest of them, highlighting the need for further studies to clarify these aspects, identify factors that may influence them, and understand how to optimize training according to different age groups. A relevant aspect concerns the interest in the prevention of HAIs was highlighted positively by 100% of the respondents to Question 20 (Figure 4). This data indicates a strong awareness among students on the importance of addressing this global healthcare issue. The total convergence on this opinion suggests that, regardless of the area of study or other variables, HAIs prevention is perceived as a crucial objective in the health and social context. This finding likely reflects a general awareness of the significant impact that HAIs have on patient well-being and the healthcare system. Different studies and guidelines have repeatedly emphasized the important role of HAIs prevention and associated preventive measures (19, 29, 30, 37, 42, 44). Furthermore, responses to Question 20.1 provided insight into the reasons behind this consensus (Figure 4). Our data strongly demonstrated that, among survey participants, there is a widespread opinion that protecting citizen’s health should be the main reason for preventing HAIs. This highlighted how students perceive the prevention of HAIs as a collective protection measure, emphasizing the importance of avoiding harm to patients, who represent the most vulnerable group in the healthcare setting. Probably this prospective may reflect a broader societal belief in the value of health as shared responsibility, even considering, for instance, that article n. 32 of the Italian Constitution protects health as a fundamental right of the individual and as a collective interest (51). Despite the awareness of the importance of preventing HAIs as a form of protecting the common good, our survey highlighted a significant lack of awareness among our respondents regarding information campaigns on HAIs. Although the majority of students recognize the need to deepen their knowledge on this topic (Question 17), only a minority (19.3%, *N* = 194) is aware of training events (Question 21), and an even a smaller number (2.6%, *N* = 26) actually participated in such initiatives (Question 22). Interestingly, among these, 8 students indicated that they had taken part in campaigns, even though they were not currently aware of any ongoing initiatives. This data suggests that their participation may refer to previous activities and is not strictly connected to currently ongoing campaigns. This limited knowledge and participation may result from several combined factors, such as the limited access to information or a reduced perception of the relevance of the topic. Another possible reason concern the use of communication channels that are not suitable for young people, as university students, such as traditional tools, which cannot compete with the attention given to social media and digital platforms. Moreover, low participation may reflect a lack of stimuli or interactive initiatives that involve students in a more direct and active way. Considering our data, this disparity is accentuated, once again, if we consider the areas of study to which the students belong, their occupation, their age, and whether they have direct experience of HAIs or in relation to the number of chronic diseases of a relative/loved one. In particular, the data indicate that students who are younger, do not belong to healthcare area, or are not employed as HCWs are less likely to be aware of the campaigns, whereas students who have had an HAI or who have a relative/loved one with a greater number of chronic diseases are more likely to be aware of the topic. This aspect highlights a need for more targeted training as suggested by different scholars (26–28, 41–44, 50). Lastly, the role of visitors in preventing HAIs was recognized by 87.2% (*N* = 873) of students (Figure 5). This role was considered particularly important by students already employed as HCWs. These results are consistent with those of other studies highlighting the importance of visitors in this context (52, 53). For instance, in Jeyasheelan et al. 87% of participants agreed that visitors could carry infectious agents that are harmful to patients (37). Again, the results of our survey suggest that healthcare education, particularly working as HCWs, and personal experiences, such as caring for a family member during a long-term hospital stay, significantly influence students’ perception of the importance of the role of visitors in preventing HAIs, increasing it. A greater propensity to consider the role of visitors as fundamental emerged among female students, a finding that aligns with some literature studies that highlight how women generally have greater knowledge about HAIs and a greater attitude to adopt proactive behaviors to prevent the transmission of these infections (28, 54). Visitor activities, such as hand washing and wearing masks, are considered very important, as already observed in Question 16. Although some practices (e.g., avoiding bringing food) are less relevant, the mean scores for these activities were generally high. Significant differences emerged between the groups of students who recognized the role of visitors as important in the prevention of HAIs and those who did not. Mann–Whitney U tests and Bonferroni adjustment showed that the ratings differed significantly for the majority of the activities analysed (*p*-adj value < 0.05), indicating that, although all students gave a fairly high rating to visitor activities, those who recognized the importance of visitors in the context of HAIs tended to give higher ratings than those who did not. These data support again the importance of association between a well-structured educational strategies and the involvement of all actors in the healthcare area (27, 41, 44, 50).

Moreover, the intention to include non-healthcare students was theoretically grounded, even though it is more expected that students enrolled in medical and healthcare programs would have a higher awareness and knowledge about HAIs, also due to their direct involvement in clinical training and infection control concepts. The entire community is involved in the issue of HAIs, which represent a broader public health concern that extends beyond the hospital setting. Every individual, such as a patient, caregiver, visitor, or member of the general population, surely has a role in infection transmission and prevention (8). For this reason, it is crucial to explore awareness and attitudes also among students from non-healthcare areas, who may not receive formal education on these topics but are nonetheless potential vectors or stakeholders in infection prevention. Raising awareness in these groups helps promote a shared responsibility culture that supports not only the idea that infection control is limited to healthcare professionals but also that it must be a societal commitment.

Another aspect to be considered concerns the different strategies, from a practical point of view, for integrating HAIs education into academic programs and beyond. For instance, it could certainly be interesting to add short modules focused on infection prevention and control during the transition from secondary school to university. Specific lessons on HAIs could also be incorporated into mandatory courses, such as compulsory courses on workplace and laboratory safety, which are already included in most degree programs. Finally, the inclusion of case studies and learning activities based on HAI-related issues could encourage not only active engagement but also critical thinking among students. This project could be extended to both healthcare courses, humanities and literature courses. In humanities and literature course, HAIs education could also be integrated through tailored approaches: for example, in law and social sciences by addressing medico-legal responsibilities and public health policies, or in communication programs by focusing on the development of accurate awareness campaigns and on how to properly inform the population about infection prevention. Furthermore, non-healthcare disciplines could have a crucial role in discussing governance aspects, such as the ethical and regulatory dimensions of infection control policies. Another important aspect concerns the understanding of how epidemiological data are managed, interpreted, and communicated to the population within public health frameworks. Even when addressed in non-healthcare contexts, HAIs, considered from a legal, communicative, or social point of view, offer students the opportunity to gain further tools for understanding and, consequently, to increase their knowledge and awareness of HAIs. These approaches could lead to greater knowledge, but can also aim to promote a culture of prevention in a more multidisciplinary and focused academic context.

To improve clinical outcomes and effectively reduce the incidence of HAIs, it has been demonstrated that not only educational but also organizational aspects must be taken into account. The aim is to ensure alignment with broader and more consistent evidence showing the effective reduction of antibiotic use and management in line with structured prevention programs. For this reason, the most effective strategies to support this behavioral change and optimize infection control were highlighted in studies that demonstrate the need to obtain a comprehensive and multi-component approach that integrates training, monitoring, and governance (55, 56). Consequently, integrating the infection prevention principles into university programs, whether in the health area or in the humanistic one, based on this evidence, could contribute to promoting both a culture of prevention itself and a sense of shared responsibility from the earliest stages of education. This study has several strengths that enhance the reliability and significance of its findings. First, the sample size was large and diverse, including 1,059 students from various academic areas such as healthcare, medicine, science, law, and humanities. This allowed for a broad understanding of different perspectives on HAIs and their prevention. It is important to include not only students belonging to medical and healthcare areas, who are expected to have greater awareness of HAIs due to their direct experience with infection control, but also students from non-healthcare areas. Since infection prevention is a shared social responsibility, understanding the level of awareness among students from legal, humanities, and scientific areas provides valuable insight into how non-clinical populations perceive these risks. Knowledge of infection transmission and prevention must be important to everyone, whether they are HCWs, hospital visitors or the general public. For this reason, promoting awareness in all disciplines aims to foster a broader culture of safety in public health. Moreover, the strength of this survey is also reflected in its careful design and validation through a pilot phase, which ensured clarity and relevance, helping in the collection of accurate and meaningful data. Furthermore, the study applied robust statistical methods, including multivariate analysis, to identify patterns and correlations between factors like academic background, age, personal experience, and the impact of the Covid-19 pandemic. However, the study also presents some limitations. The response rate was moderate (15.13%), which, although in line with similar studies, limits the generalizability of the results to the broader student population. It is also possible that students who were more interested or sensitized to the topic were more likely to participate, introducing a potential non-response bias. Furthermore, the influence of recall or social desirability bias could not be excluded due to the fact that the data were self-reported. Although these limitations are common in this type of survey-based research, it is important to note that they should be taken into account when interpreting the findings. Additionally, the cross-sectional nature of the study means that it provides a snapshot of student awareness at a single point in time, without tracking changes over time. Moreover, the study was conducted within a single institution, the University of Padua, which may limit the applicability of the findings to other academic and cultural settings. While this monocentric design allowed for rigorous data control and internal consistency, the lack of national-level data or external validation prevents direct comparison with other universities or regions. Therefore, further multicenter or nationwide studies would be useful to confirm these findings and to assess both potential geographical and institutional variations. Despite these limitations, the study provides valuable insights into HAIs awareness and highlights key areas for improving student education and engagement in infection prevention.

## 5 Conclusion

This study demonstrated a good level of awareness among students regarding HAIs and their prevention, highlighting a sense of responsibility toward public health. However, awareness and attitudes towards HAIs prevention were found to be associated with several factors, including the area of study, age, personal health experiences, and professional background. Students from medical and healthcare-related areas reported a high level of understanding and interest in HAIs prevention, whereas those from legal, humanities, and some science fields reported lower levels of engagement.

Personal experiences, such as caring for a relative/loved one during a long hospitalization, were significantly associated with higher awareness of HAIs. In addition, students declared that the Covid-19 pandemic coincided with a broadening of their awareness on infection prevention. Despite this broad awareness, knowledge gaps persist, particularly among students from non-healthcare and non-medical areas.

To address these disparities, targeted educational programs and curriculum adjustments are necessary. Tailored approaches that account for different backgrounds and influencing factors may help improve overall awareness and engagement. While some factors, such as age and personal health experiences, cannot be modified, others, such as education and training, can be strategically improved. Integrating health-related content into different academic programs and encouraging interdisciplinary learning may foster a more collaborative approach to HAIs prevention. Future research and intervention programs should focus on enhancing student knowledge and involvement across all fields, equipping them with the tools necessary to reduce the impact of HAIs on public health and the healthcare system.

## Data Availability

The raw data supporting the conclusions of this article will be made available by the authors, without undue reservation.
