# Supplementary material for: Cross-disciplinary awareness of healthcare associated infections (HAIs): insights from a university-wide survey
Source: Front Med (Lausanne). 2025 Nov 10;12:1642560. doi: 10.3389/fmed.2025.1642560 (PMC12640835; doi:10.3389/fmed.2025.1642560)
Supplement: Supplementary file 1 [file Table_1.pdf]

Supplementary Material

Supplementary Material - Table A. Full results of the multivariable logistic regression models on knowledge, attitudes, and perceptions toward HAIs, including variables, reference categories, coefficients, standard errors, p-values, odds ratios (ORs), and 95% confidence intervals (CIs).

Reference Question	Variable	Compared Category	Reference Category	Std. Error	P-value	Odds Ratio	95% CI	Interpretation
Knowledge of HAIs (Q7)	Area of Study	Legal & Humanities	Healthcare	0.36	2.11e-09	0.12	0.06-0.20	Students in legal & humanities are significantly less likely to know about HAIs than healthcare students.
	Year of Course	Progression in year	Earlier year	0.10	4.97e-05	1.53	1.26-1.90	Advancing in the course increases likelihood of being aware of HAIs.
	Relative/Loved one with chronic disease	Yes	No	0.31	1.29e-02	2.17	1.20-4.12	Having a relative with chronic disease increases knowledge of HAIs.
	Relative/Loved one cared for during hospitalization >5 days	Yes	No	0.30	2.00e-02	2.01	1.14-3.69	Caring for hospitalized relative increases knowledge of HAIs.
Feeling informed about HAIs (Q17)	Area of Study	Legal & Humanities	Healthcare	0.28	2.22e-16	0.10	0.06-0.17	Much lower probability to feel they need to explore further the topic.
	Area of Study	Legal & Humanities	Healthcare	0.44	1.75e-14	0.03	0.01-0.08	Much lower probability to feel sufficiently informed.
	Area of Study	Medical	Healthcare	0.24	3.18e-02	0.60	0.38-0.96	Medical students less likely to need to explore further compared to healthcare.
	Area of Study	Medical	Healthcare	0.26	1.39e-02	0.52	0.31-0.88	Medical students less likely to feel sufficiently informed compared to healthcare.
	Area of Study	Scientific	Healthcare	0.64	3.55e-03	0.15	0.04-0.54	Scientific students much less likely to need to explore further compared to healthcare.
	Area of Study	Scientific	Healthcare	0.79	5.14e-03	0.11	0.02-0.52	Scientific students much less likely to feel sufficiently informed compared to healthcare.
	Year of Course	Progression in year	Earlier year	0.10	2.29e-03	1.36	1.12-1.67	Progression in course increases probability of feeling sufficiently informed.
	Cared for hospitalized relative	Yes	No	0.19	2.60e-02	1.53	1.05-2.22	Caregiving increases probability of wanting to explore topic further.

**Covid-19 impact on HAIs knowledge (Q18)**	Area of Study	Legal & Humanities	Healthcare	0.30	1.03e-05	0.27	0.15-0.49	Lower probability of pandemic-driven knowledge acquisition compared to healthcare.
	Area of Study	Medical	Healthcare	0.25	4.13e-02	0.60	0.37-0.98	Lower probability of pandemic-driven knowledge acquisition compared to healthcare.
	Area of Study	Scientific	Healthcare	0.64	4.57e-02	0.28	0.08-0.98	Lower probability of pandemic-driven knowledge acquisition compared to healthcare.
	Area of Study	Legal & Humanities	Healthcare	0.53	2.95e-04	0.14	0.05-0.42	Lower probability of reporting all knowledge acquired during pandemic compared to healthcare.
**Perceived interest in HAIs (Q19)**	Age	>24 years	<21 years	0.08	6.45e-03	1.26	1.07-1.49	Older students perceive higher public interest.
	Relative with chronic disease	Yes	No	0.06	1.73e-02	1.16	1.03-1.30	Relatives with chronic disease increase perception of higher public interest.
	Area of Study	Legal & Humanities	Healthcare	0.10	1.22e-08	0.57	0.47-0.69	Lower perception of public interest compared to healthcare.
	Area of Study	Scientific	Healthcare	0.25	1.21e-02	0.54	0.33-0.87	Lower perception of public interest compared to healthcare.
**Reason for prevention importance (Q20.1)**	Graduated students	Yes	No	0.27	3.49e-03	2.21	1.29-3.78	Graduates give more importance to citizen health than cost of additional care.
	Year of Course	Progression in year	Earlier year	0.18	1.83e-03	0.58	0.41-0.82	Year progression reduces likelihood of preferring HCWs health over cost of additional care.
	Relative with chronic disease	Yes	No	0.35	2.55e-02	2.18	1.10-4.31	Relatives with chronic disease increase likelihood of preferring HCWs health over cost of additional care.
**Awareness of campaigns (Q21)**	Age quartile	>24 years	<21 years	0.25	1.53e-03	2.18	1.34-3.53	Less aware of campaigns.
	Occupation	Only Student	HCW	0.32	7.60e-06	0.24	0.13-0.45	Less aware of campaigns.
	Occupation	PhD student/Researcher	HCW	0.63	1.03e-02	0.20	0.05-0.63	Less aware of campaigns.
	Occupation	Non-HCW	HCW	0.35	2.24e-03	0.35	0.17-0.68	Less aware of campaigns.
	Area of Study	Legal & Humanities	Healthcare	0.38	1.75e-03	0.30	0.13-0.61	Less aware of campaigns.
	Relative with chronic diseases	More chronic diseases	Fewer chronic diseases	0.09	1.95e-02	1.24	1.03-1.48	Each additional chronic disease in relatives increases awareness of campaigns.
	Student experience of HAI	Yes	No	0.48	5.12e-02	2.55	0.97-6.53	Increases awareness of campaigns.

**Participation in campaigns (Q22)**	Graduated students	Yes	No	0.48	3.67e-03	0.25	0.10-0.64	Less likely to participate in campaigns.
**Visitor role in prevention (Q23)**	Occupation	Only Student	HCW	1.01	3.01e-02	0.11	0.01-0.51	Less likely to give importance to the visitors role in healthcare context.
	Occupation	PhD student/Researcher	HCW	1.15	4.05e-02	0.09	0.01-0.69	Less likely to give importance to the visitors role in healthcare context.
	Occupation	Non-HCW	HCW	1.04	2.38e-02	0.10	0.01-0.48	Less likely to give importance to the visitors role in healthcare context.
	Relative/Loved one cared for during hospitalization >5 days	Yes	No	0.20	3.52e-02	1.53	1.04-2.29	Increase likelihood to give importance to the visitors role in healthcare context.
	Gender	Male	Female	0.20	3.71e-04	0.49	0.34-0.73	Less likely to give importance to the visitors role in healthcare context.
