# Supplementary material for: Cross-disciplinary awareness of healthcare associated infections (HAIs): insights from a university-wide survey
Source: Front Med (Lausanne). 2025 Nov 10;12:1642560. doi: 10.3389/fmed.2025.1642560 (PMC12640835; doi:10.3389/fmed.2025.1642560)
Supplement: Supplementary file 2 [file Data_Sheet_1.pdf]

Supplementary Material

1.1 Translated English version of the survey on HAIs

Question 1. Age: ________________________

Question 2. Gender:

- Female
- Male
- Prefer not to answer
- Other → Question 2.1. Please specify other

Question 3. Nation: ________________________

Question 4. Occupation (multiple selections allowed):

- ☐ Only Student
- ☐ PhD student/Researcher
- ☐ Healthcare worker
- ☐ Craftsperson/Laborer
- ☐ Shopkeeper/Salesperson
- ☐ Freelancer
- ☐ Armed Forces
- ☐ Manager/Entrepreneur
- ☐ Public Employee
- ☐ Private Employee
- ☐ Unemployed
- ☐ Retired
- ☐ Other → Question 4.1. Please specify other

Question 5. Which type of high school did you attend?

- Classical High School
- Scientific High School/Applied Sciences Scientific High School
- Linguistic High School
- Human Sciences High School
- Artistic High School
- Musical and Choreutic High School
- Technical Institute (Economic sector)
- Technical Institute (Technological sector)
- Professional Institute
- Other → Question 5.1. Please specify other

Question 6.1. What degree course are you currently attending?
- Bachelor’s Degree
- Master’s Degree
- Single-cycle Master’s Degree

Question 6.2. To which area does your degree course belong?
- Healthcare Area
- Medical Area
- Veterinary Medicine Area
- Biological/Biotechnological Scientific Area
- Non-Biological/Biotechnological Scientific Area
- Legal Area
- Humanities Area
- Technological Area
- Other → Question 6.2.1. Please specify other

Question 6.3. What year of the course are you currently attending? (Enter an integer from 1 to 6)

____

Question 6.4. In which year did you enroll in your current degree course? (Ex. if Academic Year 2022/2023, write 2022) ____________

Question 6.5. Do you already have a degree?
- No
- Yes

Question 6.5.1. Indicate the field of the degree you have already obtained:
- Healthcare Area
- Medical Area
- Veterinary Medicine Area
- Biological/Biotechnological Scientific Area
- Non-Biological/Biotechnological Scientific Area
- Legal Area
- Humanities Area
- Technological Area
- Other → Question 6.5.2. Please specify other

Hospital infections, also known as nosocomial infections or healthcare-associated infections (HAIs), are those infections contracted by a patient during hospitalization or in another healthcare facility. These infections can affect individuals hospitalized for various reasons, such as surgeries, medical treatments, intensive care, or long-term stays.

Question 7. Before reading the above definition, were you aware of what HAIs were?
- No

- Yes

Question 8. Have you ever contracted a HAI?

- No
- Yes

Question 9. Have you ever had to undergo a long hospital stay (more than 5 days)?

- No
- Yes

Question 10. Have you ever been hospitalized more than 3 times in your life? (Each stay must have lasted more than 5 days)

- No
- Yes

Question 11. Do you suffer from a chronic illness?

- No
- Yes

Question 11.1. (If answer on Question 11 is "Yes") What type of chronic illness do you suffer from? (Multiple selections allowed)

- ☐ Neurodegenerative
- ☐ Respiratory
- ☐ Hematologic (non-oncological)
- ☐ Oncological/Hemato-oncological
- ☐ Cardiovascular
- ☐ Metabolic
- ☐ Autoimmune and Inflammatory
- ☐ Gastrointestinal
- ☐ Other → Question 11.1.1. Please specify other

Question 12. Has a relative/loved one of yours ever contracted a HAI?

- No
- Yes

Question 13. Have you ever had to care for a relative/loved one who faced a long hospital stay (more than 5 days)?

- No
- Yes

Question 14. Has a relative/loved one of yours been hospitalized more than 3 times? (Each stay must have lasted more than 5 days)

- No
- Yes

Question 15. Does a relative/loved one of yours suffer from a chronic illness?

- o No
- o Yes

Question 15.1. (If answer on Question 15 is “Yes”) What type of chronic illness does your relative/loved one suffer from? (Multiple selections allowed)

- ☐ Neurodegenerative
- ☐ Respiratory
- ☐ Hematologic (non-oncological)
- ☐ Oncological/Hemato-oncological
- ☐ Cardiovascular
- ☐ Metabolic
- ☐ Autoimmune and Inflammatory
- ☐ Gastrointestinal
- ☐ Other → Question 15.1.1. Please specify other

Question 16. What do you think are the main preventive measures to reduce the incidence of HAIs? (Multiple selections allowed)

- ☐ Sterilization and disinfection
- ☐ Keeping hair tied back
- ☐ Regular intake of vitamins to boost immunity
- ☐ Monitoring health status
- ☐ Hand hygiene
- ☐ Increased consumption of citrus fruits to strengthen the immune system and prevent infections
- ☐ Proper use of medical and personal devices
- ☐ Using coarse-mesh filters to purify the air

Question 17. Do you feel sufficiently informed about HAIs and their management?

- o No, I don't have much knowledge of the subject
- o Yes, but I believe I need to explore the topic further
- o Yes, I feel sufficiently informed about the subject

Question 18. Do you believe that the pandemic experience has increased your knowledge on the subject?

- o No, my current knowledge is the same as it was before the pandemic
- o Partly, the pandemic experience led me to deepen my knowledge of the subject
- o Yes, all of my knowledge comes after the pandemic experience

Question 19. On a scale of 1 to 5, where 1 indicates no interest and 5 maximum interest, what do you think is the current interest in HAIs? ____________

Question 20. Do you believe that the prevention of HAIs is important in the healthcare setting?

- No
- Yes

Question 20.1. (If answer on Question 20 is "Yes") Why do you think that the prevention of HAIs is important in the healthcare setting?

- Due to the costs on the national health system related to additional treatments
- For the health of citizens in general
- For the health of healthcare workers
- Due to the costs on the national health system related to medical-legal disputes

Question 20.1. (If answer on Question 20 is "No") Why do you think the prevention of HAIs is NOT important in the healthcare setting?

- Because resources should be allocated to treatment
- Because the risk of infections in hospitals cannot be eliminated
- Because prevention is difficult to achieve
- Because I don't think it's a priority issue to address

Question 21. Are you aware of information campaigns on HAIs?

- No
- Yes

Question 22. Have you ever participated in information campaigns on HAIs?

- No
- Yes

Question 23. When you access a hospital as a visitor/companion, do you believe you play an important role in preventing HAIs?

- No
- Yes

Question 23.1 Based on your knowledge as a visitor/companion in a hospital, indicate for each of the following activities the importance you believe they have in preventing HAIs (if you know). Where 1 means no importance and 5 means maximum importance.

- Hand washing __________
- Bringing food or beverages to the patient __________
- Bringing books, magazines, or other items to the patient __________
- Using a personal vehicle instead of public transport to reach the hospital __________
- Wearing clean clothes __________
- Wearing a mask __________
- Limiting the number of relatives/friends during visits __________
- Not using the patient's room bathroom __________
